# Evaluation of the Daily Change in PaO_2_/FiO_2_ Ratio as a Predictor of Abnormal Chest X-rays in Intensive Care Unit Patients Post Mechanical Ventilation Weaning: A Retrospective Cohort Study

**DOI:** 10.3390/medicina58020303

**Published:** 2022-02-17

**Authors:** Won-Gun Kwack

**Affiliations:** Division of Pulmonary, Allergy and Critical Care Medicine, Department of Internal Medicine, Kyung Hee University Hospital, Seoul 02447, Korea; wongunnim@naver.com; Tel.: +82-2-958-8194

**Keywords:** chest X-ray, intensive care unit, mechanical ventilation, PaO_2_/FiO_2_ ratio

## Abstract

*Background and Objectives:* The routine daily chest X-ray (CXR) strategy is no longer recommended in intensive care unit (ICU) patients. However, it is difficult for intensivists to collectively accept the on-demand CXR strategy because of the ambiguous clinical criteria for conducting CXRs. This study evaluated the predictive value of the change in PaO_2_/FiO_2_ (PF ratio) for abnormal CXR findings in ICU patients after mechanical ventilation (MV). *Materials and Methods:* A retrospective cohort study was conducted between January 2016 and March 2021 on ICU patients with MV who had at least 48 h of MV, and stayed at least 72 h in the ICU post-MV. Routine daily CXRs and daily changes in the PF ratios were investigated during the three days post-MV. *Results:* The 186 patients included in the study had a median age of 77 years (interquartile range: 65–82), and 116 (62.4%) were men. One hundred and eight (58.1%) patients had abnormal CXR findings, defined as one or more abnormal CXRs among the daily CXRs during the three days post-extubation. The reintubation rate was higher in the abnormal CXR group (*p* = 0.01). Of the 558 CXRs (normal = 418, abnormal = 140) and PF ratios, the daily change in PF ratio had a significant predictive accuracy for abnormal CXR findings (AUROC = 0.741, *p* < 0.01). *Conclusions:* The change in PF ratio (the Youden index point: ≤−23) had a sensitivity of 65.7%, and a specificity of 79.9%. Based on these results, the daily change in the PF ratio could be utilized as a predictive indicator of abnormal CXRs in ICU patients after MV treatment.

## 1. Introduction

Routine daily anterior–posterior chest X-ray (CXR) is a common practice for patients in intensive care units (ICUs) to monitor the improvement or deterioration of a disease, or to detect medical intervention-induced complications, especially in patients receiving mechanical ventilation (MV) [1]. However, previous studies and systemic reviews have reported that the diagnostic efficacy of daily routine CXRs is low. In addition, this routine CXR strategy does not improve outcomes (length of stay in the ICU, ICU mortality, and re-admission to ICU) compared to the on-demand CXR strategy [2,3,4]. Therefore, the American College of Radiology and the four major U.S. critical care societies do not currently recommend daily CXRs during critical care [5,6].

The ratio of arterial oxygen partial pressure (PaO_2_) and the fraction of inspired oxygen (FiO_2_) is widely used in ICUs as an indicator of oxygenation status, since this metric provides breathing-related data quickly and easily. A low PaO_2_/FiO_2_ (PF ratio) at ICU admission has been associated with poor ICU patient outcomes, including higher ICU mortality and longer hospital stays [7,8]. A previous study reported a correlation between PF ratio and the severity scoring of lung edema on the CXRs of critically ill patients [9].

Although multiple guidelines recommended an on-demand CXR strategy, it has not yet been adopted uniformly because of the culture around the longstanding clinical practice, and anxiety about judging the appropriate time for CXRs due to differences in the experience of clinicians. To reduce routine daily CXRs, a previous single-center study proposed organizational changes, such as the education of staff members and a restricted prescription using checklist requirements. With these changes, the number of annual CXRs could be reduced by about 50% without increasing mortality and length of ICU stay, resulting in about $200,000 in savings per year in critical care [10]. In addition to these efforts to change the practice culture, a numerical indicator that can predict abnormal CXRs could be used as an additional tool, so that the on-demand strategy can be universally accepted in the clinical field. Therefore, this study evaluated whether the PF ratio can be applied as a predictive indicator to enhance the on-demand CXRs strategy. Since the change in clinical practice can be accommodated more easily in stable patients than unstable patients requiring frequent treatment changes, this study included relatively stable patients who had finished MV treatment.

This study evaluated the correlation between the daily change in the PF ratio and aggravating CXR changes in ICU patients after weaning from ventilator support and extubation. The study aimed to estimate the predictive power of the daily change in PF ratio.

## 2. Materials and Methods

### 2.1. Study Design and Patients

A retrospective cohort study was conducted on patients undergoing MV and admitted to a medical or surgical ICU in a tertiary academic hospital between January 2015 and February 2020. The patients who received MV for ≥48 h and had a post-MV ICU stay ≥72 h were analyzed based on their electronic medical records. The patients with a tracheostomy, extubation failure (reintubation ≤ 72 h), or those lacking clinical information were excluded. The collected variables included the following: age, sex, acute physiology, chronic health evaluation (APACHE) II within 24 h of ICU admission, cause of admission (medical or surgical treatment), chronic underlying diseases, the reason for intubation, vasopressor use, mean arterial pressure, arterial blood gas analysis, CXR findings, oxygen supply tools and FiO_2_, duration of MV (pre-extubation), post-extubation sedative use, reintubation (>72 h post-extubation), duration of post-extubation ICU stay, length of total ICU stay, ICU mortality, and duration of hospital stay. Chronic underlying diseases were defined as the following: cardiovascular diseases, such as heart failure or coronary artery disease; chronic lung diseases, such as chronic obstructive pulmonary disease, asthma, or interstitial lung disease; and cerebral vascular diseases, such as cerebral infarction, cerebral hemorrhage, or Parkinson’s disease. The association between the change in the PF ratio and abnormal CXR findings was evaluated using a receiver operating characteristic (ROC) curve analysis. The Institutional Review Board (IRB) of Kyung Hee University Hospital approved this study (approval no.: 2021-03-107; date of approval: 9 April 2021), and informed consent was waived due to the retrospective study design. 

### 2.2. Routine Daily CXRs and PF Ratio

Daily routine CXR and PF ratio data on extubation day and post-extubation days 1, 2, and 3 were obtained. The routine daily tests were usually performed between 3 a.m. and 5 a.m. The difference between the time of CXR and PF ratio calculations did not exceed 1 h. During the post-extubation period, a high-flow nasal cannula was used as an oxygen supply tool. The daily changes in PF ratios were defined as the difference compared to the previous day’s value. Abnormal CXR findings included the occurrence or aggravation of infiltration, atelectasis, pleural effusion, or pulmonary edema compared to the CXRs conducted on the previous day. The CXR results were double-checked by two pulmonologists who were blinded to the other study data, including the PF ratio and the other pulmonologist’s judgment. Cases that were determined as abnormal by both pulmonologists were considered abnormal CXRs in the study data. An abnormal CXR group was defined as patients in which abnormal findings were identified in one or more CXRs after extubation.

### 2.3. Statistical Analysis

The statistical power of this study was calculated, and continuous variables were expressed as a mean and standard error or median and interquartile range (IQR). Categorical data were presented as numbers and percentages. The continuous variables were compared using a Student’s *t*-test or Mann–Whitney U test, and the categorical variables were compared using a chi-squared test. The correlation between the change in PF ratio and CXR abnormalities was quantified using multiple logistic regression analysis. The adjusted variables included age, sex, APACHE II, PF ratio on extubation day (pre-extubation state), post-extubation vasopressor usage, and post-extubation sedative usage. The accuracy of the change in PF ratio for predicting CXR abnormalities was quantified using the area under the ROC curve (AUROC), and the Youden index point was calculated as the optimal threshold for the ROC analysis [11]. AUROC represents the degree of separability that indicates the ability of the model to distinguish between classes. The Yuden index indicates the point at which the sum of the sensitivity and specificity is at a maximum on the ROC. The statistical power of this study was 0.82 for multiple logistic regression analysis, and 0.99 for ROC analysis. The sensitivity, specificity, positive predictive values (PPV), negative predictive values (NPV), positive likelihood ratio (PLR), and negative likelihood ratio (NLR) (according to cut-off values of the change in PF ratio) were also calculated to assess the cut-off values. SPSS 23.0 for Windows (SPSS, Chicago, IL, USA) and MedCalc 19.8 for Windows (MedCalc, Ostend, Belgium) were used for the statistical analyses, and *p*-values of less than 0.05 were considered significant.

## 3. Results

**Figure 1:**
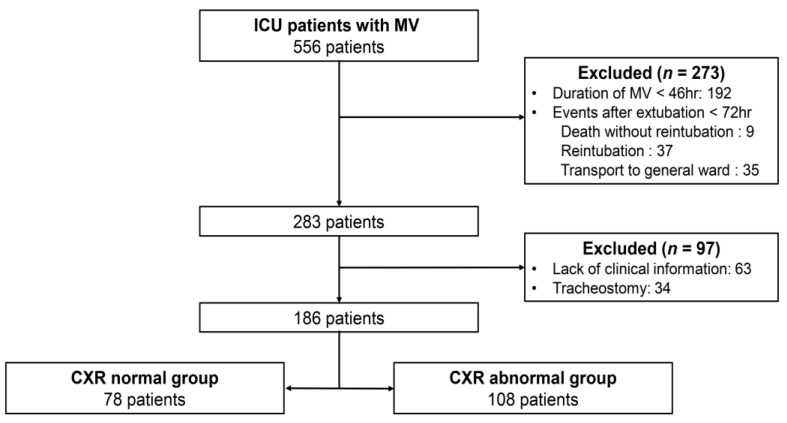
Flow diagram of patient selection. The CXR abnormal group included cases in which abnormal findings were observed in one or more CXRs on sequential CXRs after extubation. CXR = chest X-ray, ICU = intensive care unit, MV = mechanical ventilation.

During the study period, 556 patients that received MV were admitted to the medical or surgical ICU. Of these patients, 370 were excluded based on the following exclusion criteria: duration of mechanical ventilation <48 h (192 patients), reintubation and duration of post-extubation ICU stay <72 h (81 patients), lack of clinical information (63 patients), and tracheostomy (34 patients). In total, 186 participants were included in the study (Figure 1). The median age of the enrolled patients was 77 years (IQR 65–82), and 116 (62.4%) were men. The mean APACHE II score was 14.16 ± 0.36, and 140 (75.3%) were medical patients. A total of 558 daily CXRs (normal = 418, abnormal = 140) and PF ratios were analyzed to determine the correlation.

The general characteristics of the subjects at the time of ICU admission according to post-extubation CXR findings are summarized in Table 1. There were no statistically significant differences in age, sex, and underlying diseases between the CXR normal and abnormal groups. In addition, there were no significant differences in APACHE II score, PF ratio, or reason for intubation between the groups (*p* = 0.77, 0.81, and 0.76, respectively).

As shown in Table 2, there were no statistically significant differences between the CXR normal and abnormal groups in terms of laboratory results, PF ratio, or the duration of MV before extubation. Reintubation was more frequent in the CXR abnormal group than the CXR normal group (*p* = 0.01). The duration of post-extubation ICU stay was not significantly different between the two groups. Although there was no statistical significance, the ICU mortality rate was higher in the abnormal CXR group (*p* = 0.08). 

**Figure 2:**
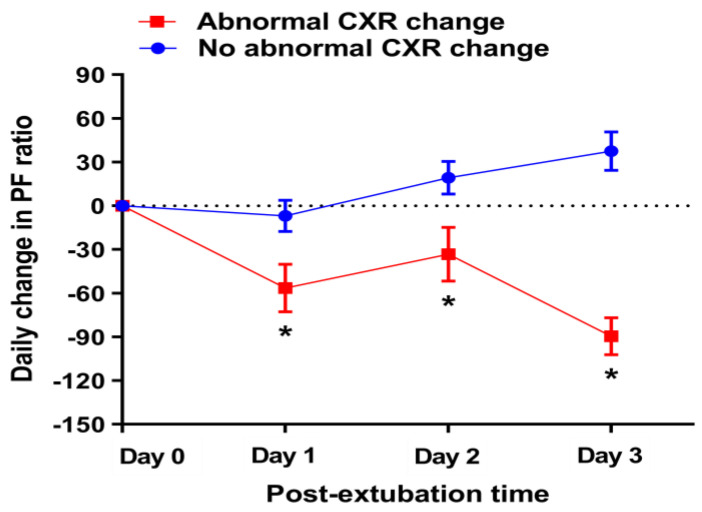
Comparison of daily change in PF ratios according to CXR findings by observation date. Values are presented as means with standard error. The number of abnormal CXR cases by observation date after extubation were the following: day 1 = 52, day 2 = 45, day 3 = 43, total = 186. * *p* < 0.01. CXR = chest X-ray, PF ratio = PaO_2_/FiO_2_.

**Figure 3:**
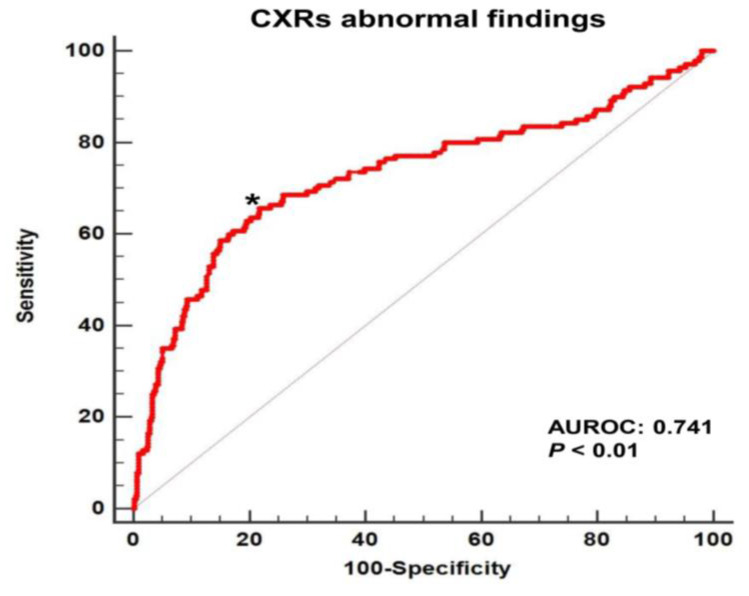
The area under the ROC curve of the daily change in PF ratio for abnormal CXRs after extubation and mechanical ventilation. * = The Youden index cut-off of −23. AUROC = area under the receiver operating characteristic curve, CXR = chest X-ray, PF ratio = PaO_2_/FiO_2_.

When comparing the daily change in PF ratios of the two groups by observation date, the patients with abnormal CXRs had significant negative changes in PF ratios compared to patients with normal CXRs (*p* < 0.01) (Figure 2). Multiple logistic regression analysis showed that the daily change in PF ratio was negatively associated with abnormal CXR findings (odds ratio = 0.990, 95% CI = 0.987–0.992, *p* < 0.01) (see online Supplementary Materials, Table S1). In the ROC curve analysis for the 558 CXRs and the daily change in PF ratios (Figure 3), the AUROC of the change in PF ratio for the abnormal CXR findings was 0.741 (*p* < 0.01). Additionally, predictive values, including sensitivity, specificity, PPV, NPV, PLR, and NLR, were assessed for the abnormal CXR findings (Table 3). The Youden index cut-off was a PF ratio ≤ −23, which had a sensitivity, specificity, PPV, and NPV of 65.7%, 79.9%, 52.3%, and 87.4%, respectively. The PPV and NPV values indicate that CXR exacerbation was observed in 65.7% of cases where the daily PF ratio change was −23 or less, and there was no CXR exacerbation in 79.9% of cases exceeding a PF ratio change of −23. The highest sensitivity (77.1%) with a specificity > 50% was observed at a cut-off of a change in PF ratio ≤ 12.

## 4. Discussion

The daily change in PF ratios demonstrated a strong correlation and significant prediction accuracy for abnormal CXRs during the post-extubation periods. The Youden index cut-off for abnormal CXRs was a change in PF ratio ≤ −23. To the best of our knowledge, this is the first study to evaluate the daily change in PF ratio as a predictor for abnormal CXRs in ICU patients after MV.

Until 2008, the routine daily CXR strategy was considered the “most appropriate” strategy for patients receiving MV [12]. However, several studies revealed that this strategy exhibited a low diagnostic efficacy (4.4%), and was not significantly correlated with patient outcomes such as ICU mortality, length of ICU stay, and duration of MV [3,13,14]. As of 2011, the daily routine CXR regiment was deemed no longer beneficial for MV patients. Since 2014, it has not been recommended as necessary for all ICU patients, particularly stable patients. Based on these guidelines, the number of ICUs that implement on-demand CXR strategies is gradually increasing. A Dutch study reported that the rate of routine daily CXRs in ICUs decreased from 63% to 7% [15,16]. However, the proportion of French ICUs using routine daily CXRs was 25–37% [17,18], and 60% of U.S. patients receiving MV from 2008 to 2014 received routine daily CXRs [6]. Although evidence-based practices (EBP) have been widely incorporated into routine practices in healthcare, there are various barriers for some treatments with confirmed efficacy to be actively accepted into clinical practice, and to improve the quality of health service. Only approximately half of all EBPs ever reach widespread clinical usage [19]. To address this issue, implementation science has been used to study and promote the systematic uptake of research findings in clinical settings. In these studies, one of the barriers to high-quality EBP utilization is the lack of cognitive transformation flexibility in healthcare providers. To mitigate this challenge, approaches such as education, prescription system changes, and audit feedback have been utilized [19,20].

The on-demand CXR strategy is considered the recommended practice for ICU patients. A questionnaire study showed that most clinical staff members (73%) thought that the routine daily CXR strategy in ICU patients should be replaced with the on-demand CXR protocol to save medical resources, and avoid unnecessary exposure to radiation [21]. Additionally, most routine daily CXRs are performed with one portable device, and the occurrence of multidrug-resistant strains of infections is gradually increasing in intensive care. Therefore, the on-demand strategy could help reduce the spread of infection following routine CXRs [22]. In the intensive care setting, the clinician’s concern for patient deterioration is likely to be higher than during the treatment of patients in the general ward. Therefore, some clinicians are more likely to perform daily routine CXRs, which they may consider safer than taking on the daily burden of determining the need for CXRs, and potentially making errors. In this situation, it is possible to improve the provider’s awareness, and successfully apply the on-demand CXR strategy through systematic education or changes in the treatment environment. However, since the available medical resources vary by treatment centers, a comprehensive approach such as this may not always be feasible. Accordingly, identifying simple and objective markers that are predictive of CXR abnormalities will provide valuable information for confirming the clinical need for CXRs. The PF ratio is expected to meet these criteria, and help promote safer and widespread adoption of on-demand CXR strategies.

In this study, daily changes in the PF ratio exhibited significant predictive accuracy for abnormal CXR findings in the post-extubation period. This result does not suggest that daily changes in the PF ratio should be the single or absolute criterion for CXRs in ICU patients. It is essential to consider the patient’s overall clinical status, including consciousness, work of breathing, and subjective symptoms. However, because the clinical proficiency and experience of attending physicians varies, numerical markers, such as the daily change in PF ratio, may be particularly useful for clinical application. Therefore, the PF ratio is proposed as an auxiliary tool for decision-making for ICU patients after extubation. This study investigated the performance of cut-off values of the daily change in PF ratio for predicting abnormal CXR findings. With a change in PF ratio cut-off of ≤−23, the specificity (79.9%) was higher than the sensitivity (65.7%), and the PLR and NLR were 2.95 and 0.44, respectively. In contrast, when the change in PF ratio cut-off was ≤12, the sensitivity (77.1%) was higher than the specificity (50.9%), and the PLR and NLR values were 1.57 and 0.45, respectively. Based on these results, a cut-off value of −23 could be used as a rule-in point, and a cut-off value of 12 could be used as a rule-out point [23].

As mentioned above, the cut-off for PF ratio change should not be prioritized over changes in other clinical statuses. Even if the on-demand CXR strategy using the change in PF ratio cut-off could reduce unnecessary CXR orders, there are also limitations to utilizing this parameter. The PF ratio usually changes in steps of delta-hundred in severe cases. Accordingly, application of this value for efficient CXR orders is thought to be more effective in clinically stable patients with little change in FiO_2_. Additionally, measuring the PF ratio requires specific oxygen supply tools that can accurately predict the FiO_2_, such as a high-flow nasal cannula or venturi mask after extubation, and involves the risk of complications due to invasive procedures or maintaining an arterial catheter for blood gas analysis. The SF ratio, which has the advantages of being non-invasive and continuous, could be an alternative indicator to consider. It is highly likely that it would be useful in patients with severe hypoxemia, since SpO_2_ has a small change range at high values of 97% or more [24].

The abnormal CXR group had a higher rate of reintubation compared to the normal CXR group. However, there were no significant differences in the durations of the total and post-extubation ICU stays. Although there was no statistically significant difference in mortality rate, the higher ICU mortality rate in the abnormal CXR group may have resulted in similar lengths of ICU stays for the two groups. In the future, large-scale studies based on critical care databases should be used to generalize and validate the predictive accuracy of the change in PF ratio. In addition, a well-controlled comparative study for co-factors should be conducted on clinical outcomes, such as mortality or duration of ICU stay, after applying the on-demand CXR strategy according to the cut-off for changes in PF ratio.

The present study had several limitations. First, it is possible that the gradual daily change in PF ratio for 2 to 3 days may be related to abnormal CXR findings. In this study, the AUROC of the change in PF ratio from the time of extubation was 0.663 (*p* < 0.01) for the abnormal CXR group (see online Supplementary Materials, Figure S1). Because of this, a future study is needed to evaluate the relationship between the change in PF ratio and CXR findings at the same time point after MV. Secondly, since this was a single-center retrospective study targeting ICU patients with MV treatment, data were only analyzed for three days after extubation to include the maximum number of patients who were tested daily. Nevertheless, the sample size was relatively small, and the patients were primarily elderly. Additionally, other factors that affect the predictive ability of the daily change of PF ratio for abnormal CXR findings may not be determined on a daily basis. These may include C-reactive protein, neurologic state, and the severity of underlying diseases. Third, although the high-flow nasal cannula generally supplies an accurate amount of oxygen, it also has inaccuracies due to variations in the patients’ airway structure or breathing behavior. Fourth, the on-demand CXR strategy can have disadvantages, such as increased labor intensity for clinicians who have to decide when to perform CXRs, the difficulty of scheduling the test, and inefficiencies due to test delays, especially in environments where resources for portable CXRs are scarce, such as radiologic technologists and portable CXR devices. It is also necessary to implement a system that can effectively handle additional CXRs that were not conducted in the morning.

## 5. Conclusions

The daily change in the PF ratio demonstrated significant predictive accuracy for abnormal CXRs in ICU patients after MV treatment. A cut-off value of −23 could potentially be utilized to predict abnormal CXR findings. Extensive, well-controlled studies are needed to provide a useful predictive marker for safer and more comfortable application of on-demand CXR strategies in intensive care.

## Figures and Tables

**Table 1:** General characteristics (at time of ICU admission) of intensive care unit patients who received mechanical ventilation.

Variables	CXR Normal Group (*n* = 78)	CXR Abnormal Group (*n* = 108)	*p*-Value
Age, years, median (IQR)	77 (61–82)	76 (68–82)	0.09
Male	51 (65.4)	65 (60.2)	0.47
Cardiovascular diseases	13 (16.7)	11 (10.2)	0.19
Chronic lung diseases	15 (19.2)	15 (13.9)	0.33
Cerebral vascular diseases	12 (15.4)	15 (13.9)	0.78
APACHE II score	14.28 ± 0.53	14.07 ± 0.48	0.77
Vasopressor use	27 (34.6)	45 (41.7)	0.33
Mean blood pressure, mmHg	86.23 ± 2.02	89.11 ± 1.70	0.27
Respiratory rate, breath/min	21.07 ± 0.41	21.74 ± 0.57	0.33
Body temperature, °C	36.49 ± 0.17	36.66 ± 0.07	0.31
O_2_ supply, FiO_2_	0.53 ± 0.03	0.56 ± 0.02	0.49
PaO_2_/FiO_2_	245.61 ± 15.64	239.53 ± 19.53	0.81
PaCO_2_, mmHg	42.08 ± 1.76	41.42 ± 1.24	0.75
WBC, ×10^3^/μL	13.18 ± 7.28	13.43 ± 1.24	0.88
Hemoglobin, g/dL	11.80 ± 0.25	11.24 ± 0.29	0.17
CRP, mg/dL	8.31 ± 1.10	9.80 ± 1.00	0.32
Creatinine, mg/dL	1.44 ± 0.17	1.25 ± 0.11	0.34
Reason for intubation			
Acute respiratory failure	60 (76.9)	81 (75.0)	0.76
Postoperative	18 (23.1)	27 (25.0)	0.76

Values are presented as mean ± standard error or number (%). The CXR abnormal group was defined by cases in which abnormal findings were observed in one or more CXRs on sequential CXRs after extubation. APACHE = acute physiology and chronic health evaluation, CRP = C-reactive protein, CXR *=* chest X-ray, FiO_2_ = fraction of inspired oxygen, IQR = interquartile range, PaO_2_ = partial pressure of oxygen in arterial blood, PaCO_2_ = partial pressure of carbon dioxide in arterial blood, WBC = white blood cell.

**Table 2:** Comparison of characteristics of the normal and abnormal CXR groups on the extubation day and post-extubation period.

Variables	CXR Normal Group (*n* = 78)	CXR Abnormal Group (*n* = 108)	*p*-Value
Extubation Day (Pre-Extubation State)			
WBC, ×10^3^/μL	10.47 ± 0.45	11.41 ± 0.55	0.19
Hemoglobin, g/dL	10.15 ± 0.20	10.74 ± 0.96	0.60
Creatinine, mg/dL	1.13 ± 0.14	1.11 ± 0.12	0.91
CRP, mg/dL	6.25 ± 0.67	8.08 ± 0.62	0.05
O_2_ supply, FiO_2_	0.32 ± 0.01	0.33 ± 0.01	0.57
PaO_2_/FiO_2_	292.90 ± 13.55	288.42 ± 9.26	0.78
PaCO_2_, mmHg	37.75 ± 0.91	36.93 ± 0.69	0.46
pH	7.46 ± 0.01	7.46 ± 0.01	0.84
Duration of MV, days	8.60 ± 0.78	7.69 ± 0.62	0.64
Post-extubation vasopressor use	14 (17.9)	18 (16.7)	0.82
Post-extubation sedative use	13 (16.9)	15 (13.9)	0.58
Reintubation	5 (6.6)	23 (21.5)	0.01
Post-extubation ICU stay, days	9.35 ± 1.20	9.56 ± 0.99	0.89
The total length of ICU stay, days	18.21 ± 1.57	17.62 ± 1.22	0.77
ICU mortality	4 (5.1)	15 (13.9)	0.08

Values are presented as mean ± SE or number (%). Reintubation was defined as cases when the patient was reintubated after 72 h post-extubation. CRP = C-reactive protein, CXR = chest X-ray, FiO_2_ = fraction of inspired oxygen, ICU = intensive care unit, MV = mechanical ventilation, PaO_2_ = partial pressure of oxygen in arterial blood, PaCO_2_ = partial pressure of carbon dioxide in arterial blood, WBC = white blood cell.

**Table 3:** Predictive values of the daily change in the PaO_2_/FiO_2_ ratio for the abnormal chest X-ray findings.

Variables	Sensitivity	Specificity	PPV	NPV	PLR	NLR
PF ratio ≤ −23 *	65.7 (57.2–73.5)	79.9 (75.7–83.6)	52.3 (44.6–59.8)	87.4 (83.6–90.6)	3.27 (2.6–4.1)	0.43 (0.3–0.5)
PF ratio ≤ −10	69.3 (60.9–76.8)	70.6 (66.0–74.9)	44.1 (37.4–50.9)	87.3 (83.2–90.6)	2.26 (1.9–2.7)	0.44 (0.3–0.6)
PF ratio ≤ 12	77.1 (69.3–83.8)	50.9 (46.0–55.8)	34.5 (29.2–40.1)	86.9 (82.1–90.9)	1.57 (1.4–1.8)	0.45 (0.3–0.6)
PF ratio ≤ 20	80.0 (72.4–86.3)	44.7 (39.9–49.6)	32.7 (27.7–37.9)	87.0 (81.7–91.2)	1.45 (1.3–1.6)	0.45 (0.3–0.6)

Sensitivity, specificity, PPV, and NPV are presented as percentages (95% CIs). PLR and NLR are presented as ratios (95% CIs). CI = confidence interval, PPV = positive predictive value NPV = negative predictive value, PLR = positive likelihood ratio, NLR = negative likelihood ratio, PF ratio = PaO_2_/FiO_2_. * Youden index cut-off.

## Data Availability

All the data used to support the findings of this study are available from the corresponding author upon reasonable request.

## Supplementary Materials

The following supporting information can be downloaded at: https://www.mdpi.com/article/10.3390/medicina58020303/s1; Table S1: Multivariate analyses of predictive values for abnormal chest X-ray findings in the post-extubation patients, Figure S1: The area under the receiver operating characteristics curve of the change in PF ratio from the time of extubation for abnormal CXR findings after extubation. Four-hundred and nineteen CXRs and change of PF ratios were used for analysis. *, The Youden index cutoff of −41. AUROC, area under the receiver operating characteristic curve; CXR, chest X-ray; PF ratio, PaO_2_/FiO_2_.
