# Supplementary material for: Evaluation of the Daily Change in PaO2/FiO2 Ratio as a Predictor of Abnormal Chest X-rays in Intensive Care Unit Patients Post Mechanical Ventilation Weaning: A Retrospective Cohort Study
Source: Medicina (Kaunas). 2022 Feb 17;58(2):303. doi: 10.3390/medicina58020303 (PMC8876640; doi:10.3390/medicina58020303)
Supplement: Supplementary file 1 [file medicina-58-00303-s001.zip › medicina-1542079-supplementary.pdf]

Evaluation of the Daily Change in PaO_2/FiO_2 Ratio as a Predictor of Abnormal Chest X-rays in Intensive Care Unit Patients Post Mechanical Ventilation Weaning: A Retrospective Cohort Study

Won-Gun Kwack

Division of Pulmonary, Allergy and Critical Care Medicine, Department of Internal Medicine, Kyung Hee University Hospital, Seoul, Republic of Korea.

Table S1. Multivariate analyses of predictive values for abnormal chest x-ray findings in the post-extubation patients

Variables	CXR abnormal (*n* = 140)		
	OR	95% CI	*P* value
Daily change in PF ratio	0.990	0.987-0.992	0.01
Age	1.010	0.993-1.028	0.24
Male	0.893	0.577-1.381	0.61
APACHE II score	0.997	0.955-1.041	0.89
PF ratio at extubation day (pre-extubation state)	0.997	0.994-0.999	0.01
Vasopressor use (post-extubation state)	1.069	0.611-1.869	0.82
Sedative use (post-extubation state)	0.714	0.388-1.316	0.28

Total number of chest x-rays is 558. *APACHE* acute physiology and chronic health evaluation, *CI* confidence interval, *CXR* chest x-ray, *OR* odd ratio *PF ratio* PaO_2/FiO_2.

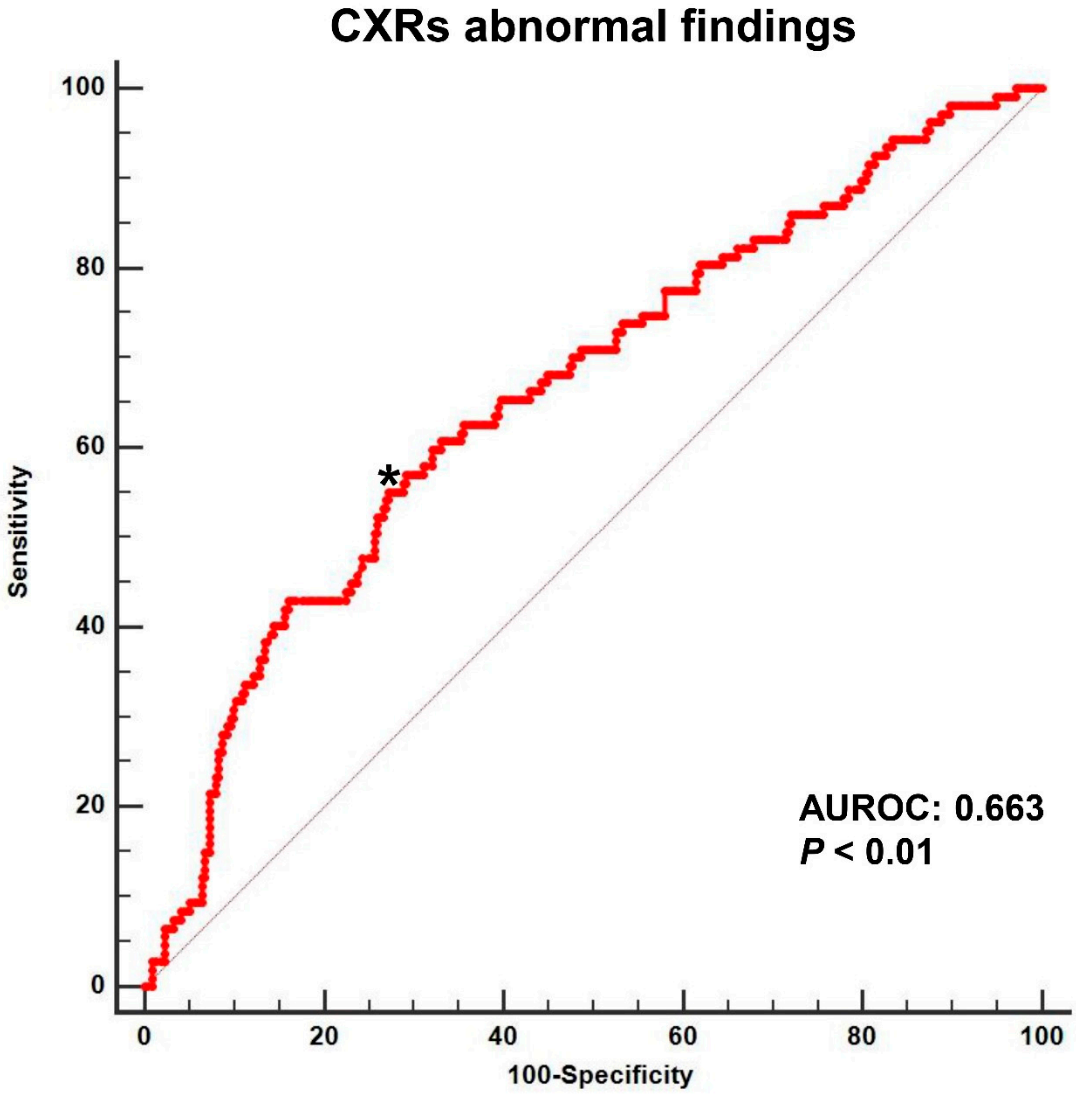

Figure S1. The area under the receiver operating characteristics curve of the change in PF ratio from the time of extubation for abnormal CXR findings after extubation. 419 CXRs and change of PF ratios were used for analysis. *; The Youden index cutoff of -41. *AUROC* area under the receiver operating characteristic curve, *CXR* chest x-ray, *PF ratio* PaO_2/FiO_2.
